# Comparison of clinical features and outcomes between HBV-related and non-B non-C hepatocellular carcinoma

**DOI:** 10.1186/s13027-020-0273-2

**Published:** 2020-02-14

**Authors:** Xiulan Xue, Wei Liao, Yugang Xing

**Affiliations:** 1 Department of Infectious Diseases, First Affiliated Hospital of Xiamen University, Xiamen, Fujian Province China; 2 Intensive Care Unit, Sun Yat-sen University Cancer Center, Guangzhou, China; 3 Department of Oncology, Kunshan Traditional Chinese Medicine Hospital, Jiangsu Province, China

**Keywords:** Hepatitis B virus, Prognosis, Hepatocellular carcinoma, Biomarker

## Abstract

**Objective:**

To evaluate the difference between hepatitis B virus related hepatocellular carcinoma (HBV-HCC) and non-HBV non-HCV hepatocellular carcinoma (NBNC-HCC) patients based on clinical features and prognosis.

**Methods:**

A total of 175 patients with HCC were enrolled. Patients’ characteristics were extracted from medical records. Among them, 107 patients were positive for HBsAg and negative for HCV-Ab while 68 patients were negative for HBsAg and HCV-Ab.

**Results:**

The patients in the NBNC-HCC group were significantly older than those in the HBV-HCC group (*P* = 0.045). Moreover, vascular invasion was found in 23.4% of HBV-HCC patients, which was significantly higher than that in the NBNC-HCC patients with 10.3% (*P* = 0.029). Kaplan-Meier analysis revealed that HBV-HCC patients had significantly worse outcomes in terms of overall survival (*P* = 0.036). Compared with the NBNC-HCC patients, the HBV-HCC patients had a significantly worse disease-free survival (*P* = 0.0018). The multivariate analysis results indicated that TNM stage (HR = 1.541, 95%CI 1.072–2.412, *P* = 0.002) and HBV infection (HR = 1.087, 95%CI 1.012–1.655, *P* = 0.042) were independent risk variables for overall survival. While vascular invasion (HR = 1.562, 95%CI 1.013–2.815, *P* = 0.042) and HBV infection (HR = 1.650, 95%CI 1.017–2.676, *P* = 0.037) were independent risk factors associated with disease-free survival.

**Conclusion:**

Our data revealed that HBV-HCC is more common in young males with vascular invasion, while NBNC-HCC occurs mostly in elderly patients, and overall survival rate is significantly better than that of HBV-HCC. Our study therefore provides evidence that patients with HBV-HCC require closer follow-up due to their poor prognosis.

## Introduction

Hepatocellular carcinoma (HCC) is one of the most common malignancies. Approximately 437,000 people are diagnosed with HCC each year, of which approximately 50% belong to East Asia [1–3]. Although treatment of HCC has improved over the decades, the 5-year survival rate of HCC patients is still low with about only 26% of HCC patients surviving [1–5]. The most prominent causes associated with HCC include chronic infection with hepatitis B virus (HBV) and hepatitis C virus (HCV) [6–9]. Although HBV-associated HCC is the largest proportion of patients with HCC in East Asia, the proportion of HCC cases without hepatitis virus surface antigen (HBsAg) and hepatitis C antibody (HCV-Ab) positive (NBNC-HCC) is growing rapidly [3–5, 10]. The background and molecular mechanisms of NBNC-HCC are unclear. However, nonalcoholic steatohepatitis (NASH) and metabolic syndrome are considered important risk factors for these patients [11–14]. Although the most important risk factors for HCC are HBV and HCV, the alcohol consumption and exposition to aflatoxin B1 are also important risk factors for HCC [15, 16]. Chronic alcohol abuse and aflatoxin B1 exposure have been widely described as two of the leading risk factors of HCC. The annual HCC rate among Child Pugh Class A or B alcoholic cirrhosis is about 2.5% and the urinary excretion of aflatoxin metabolites have been described associated with a 4 fold increase in HCC risk [15, 16].

Previous studies have compared the clinicopathological features and prognosis of HCC patients with HBV and HCV infections [17–19]. However, both of them are caused by chronic viral infection. The etiology of NBNC-HCC is mostly a metabolic factor [20, 21]. The tumor physiological characteristics are quite different from HCC caused by HBV and HCV. Several studies have compared NBNC-HCC patients with virus-related HCC patients with inconsistent results, possibly due to differences in demographic and tumor factors, and the number of patients in the cohort may be insufficient [22–24].

To further verify the difference between HBV-related HCC (HBV-HCC) and NBNC-HCC patients, we analyzed the clinical features and prognosis to provide potential evidence for different treatment strategies for HCC patients.

## Subjects and methods

### Subjects

This was a retrospective study. Patients’ characteristics were extracted from medical records. In our study, patients were included if they (1) had pathologically confirmed HCC (2) underwent hepatectomy, and (3) have medical data for hepatitis viral infection status of HBsAg and HCV-Ab. Patients were excluded if (1) they had an additional carcinoma, (2) they were HCV-Ab positive, or (3) their clinical data were not complete. To control the bias, patients were included in the study consecutively. The flow chart is shown in Additional file 1: Figure S1. A total of 175 patients with HCC, who underwent hepatectomy were enrolled. Among them, 107 patients were positive for HBsAg and negative for HCV-Ab for at least 6 months [23]. A total of 68 cases were negative for HBsAg and negative for HCV-Ab. The study was approved by the medical ethics committee. In our study, HCC was diagnosed with pathological evidences and the stage of HCC was determined according to the TNM classification. TNM staging was defined according to the American Joint Committee on Cancer TNM Staging for Liver Tumors as follows: (1) Primary tumor (T): (TX) Primary tumor cannot be assessed; (T0) no evidence of primary tumor; (T1) solitary tumor without vascular invasion; (T2) solitary tumor with vascular invasion or multiple tumors less than 5 cm in size; (T3a) multiple tumors more than 5 cm in size; (T3b) single tumor or multiple tumors of any size, involving a major branch of the portal vein or hepatic vein; and (T4) tumor(s) with direct invasion of adjacent organs, other than the gallbladder, or with perforation of visceral peritoneum. (2) Regional lymph nodes (N): (NX) regional lymph nodes cannot be assessed; (N0) no regional lymph node metastasis and (N1) regional lymph node metastasis. (3) Distant metastasis (M): (M0) no distant metastasis and (M1) distant metastasis.

### Clinical features

Clinical and pathological data on all patients, including age, gender, preoperative alpha-fetoprotein (AFP), tumor size, tumor number, capsule, and tumor differentiation were collected. Follow-up was conducted using telephonic interview. The postoperative survival of the two groups was observed. The effects of HBV- and NBNC- on the clinicopathological features and prognosis of HCC were analyzed.

### Statistical analysis

Statistical analysis was performed using the SPSS software (version 13; SPSS Inc., Chicago, IL, USA). The Student’s t test and Chi square test were used to examine the correlation between different etiologies and clinical and pathological variables. The Kaplan-Meier method (logarithmic rank test) was used to construct the survival curve. Multivariate Cox proportional hazards regression model was used to assess the independence of etiology in prediction results. *P*-values less than 0.05 were considered statistically significant.

## Results

### Association of etiology with HCC clinical features

To determine the differences between the potential clinical features of NBNC-HCC and HBV-HCC, the relationship between etiology and the clinical features of patients with HCC was evaluated. The results showed that NBNC-HCC patients were significantly older than patients with HBV-HCC (*P* = 0.045). Moreover, vascular invasion was found in 23.4% of HBV-HCC patients, which was significantly higher than that in NBNC-HCC patients with 10.3% (*P* = 0.029), as shown in Table 1.

**Table 1:** Clinical variables difference between NBNC-HCC and HBV-HCC

Variable	Etiology		*P* value
	NBNC-HCC	HBV-HCC	
Sample size	68	107	
Age, years	50.72 ± 11.90	47.09 ± 11.36	0.045
AFP, ng/mL	13,734.7 ± 46,337.2	10,729.2 ± 23,103.5	0.570
Gender			0.192
Male	58 (85.3%)	98 (91.6%)	
Female	10 (14.7%)	9 (8.4%)	
Cirrhosis			0.408
Yes	56 (82.4%)	93 (86.9%)	
vNo	12 (17.6%)	14 (13.1%)	
Tumor size, cm			0.960
< 5	11 (16.2%)	17 (15.9%)	
≥ 5	57 (83.8%)	90 (84.1%)	
Differentiation			0.313
Well-moderate	42 (61.8%)	74 (69.2%)	
Poor-undifferentiated	26 (38.2%)	33 (30.8%)	
TNM stage			0.322
I–II	37 (54.4%)	50 (46.7%)	
vIII–IV	31 (45.6%)	57 (53.3%)	
Vascular invasion			0.029
Yes	7 (10.3%)	25 (23.4%)	
No	61 (89.7%)	82 (76.6%)	

Abbreviations: NBNC:Non-HBV,Non-HCV-HCC; *HBV* hepatitis B virus

### Association of etiology with clinical outcomes in patients with HCC

**Fig. 1:**
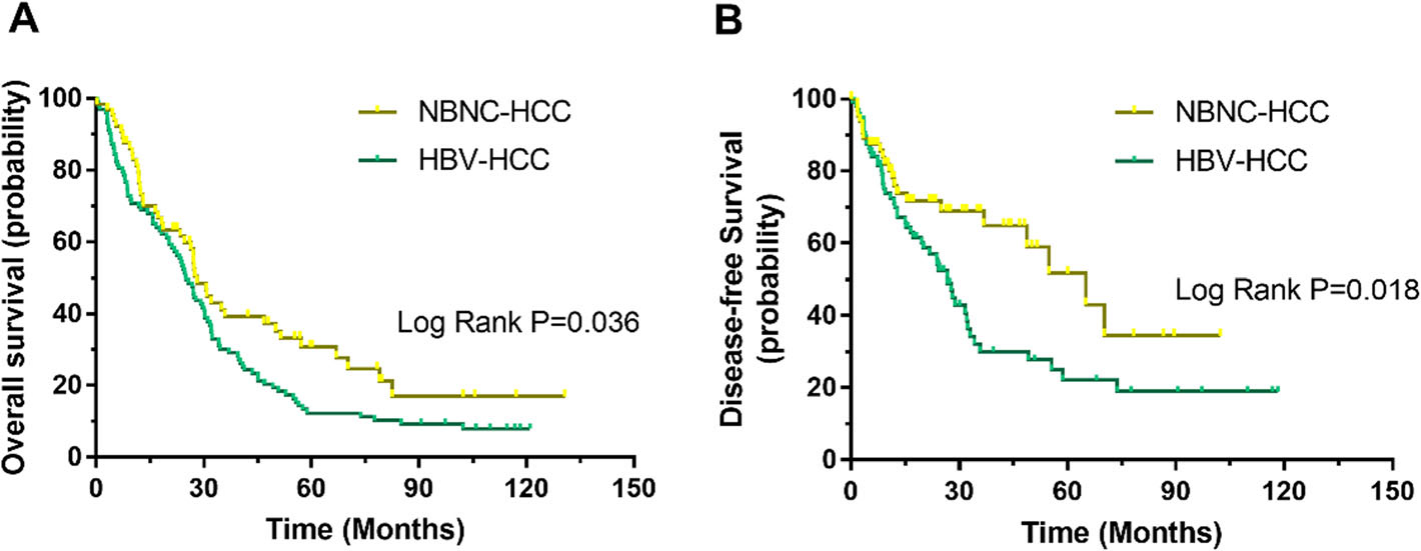
Different prognosis among patients with NBNC-HCC and HBV-HCC. **a.** Kaplan-Meier analysis revealed that HBV-HCC significantly worse outcomes in terms of overall survival (*P* = 0.036). **b.** Compared with the patients of NBNC-HCC, those with HBV-HCC had a significantly worse disease-free survival (*P* = 0.0018)

To determine the differences in etiologies and clinical outcomes among HCC patients, we conducted a Kaplan-Meier survival analysis. For the HBV-HCC patients, the Kaplan-Meier analysis revealed that they had significantly worse outcomes in terms of overall survival (*P* = 0.036). Similarly, compared with the NBNC-HCC patients, HBV-HCC patients had a significantly worse disease-free survival (*P* = 0.0018), as shown in Fig.1.

### Univariate and multivariate analyses of prognostic variables in HCC

To evaluate whether HBV infection was an independent risk factor for outcomes in HCC, both univariate and multivariate analyses were conducted. The TNM stage (*P* = 0.003), vascular invasion (*P* = 0.002), and HBV infection (*P* = 0.013) were all shown to be prognostic variables for overall survival in patients with HCC. In the multivariate analysis, only TNM stage (HR = 1.541, 95%CI 1.072–2.412, P = 0.002) and HBV infection (HR = 1.087, 95%CI 1.012–1.655, *P* = 0.042) were found to be independent prognostic variables for overall survival (Table 2).

**Table 2:** Univariate and multivariate analyses of variables for overall survival

Variables	Univariate analysis			Multivariate analysis		
	HR	95% CI	P	HR	95% CI	*P*
Age, years	1.015	0.702–1.468	0.936			
Sex	0.586	0.313–1.098	0.095			
AFP	0.842	0.570–1.244	0.387			
Cirrhosis	0.732	0.442–1.213	0.226			
Tumor size, cm	0.997	0.637–1.560	0.988			
Differentiation	1.327	0.799–2.205	0.274			
TNM stage	1.847	1.027–2.509	0.003	1.541	1.072–2.412	0.002
Vascular invasion	1.592	1.284–2.216	0.002			
HBV infection	1.372	1.040–2.003	0.013	1.087	1.012–1.655	0.042

Abbreviations: NBNC:Non-HBV,Non-HCV-HCC; *HBV* hepatitis B virus

We further explored the risk factors associated with disease-free survival (Table 3). Univariate analysis showed that vascular invasion (*P* = 0.024) and HBV infection (*P* = 0.026) were risk factors associated with disease-free survival. In the multivariate analysis, vascular invasion (HR = 1.562, 95%CI 1.013–2.815, *P* = 0.042) and HBV infection (HR = 1.650, 95%CI 1.017–2.676, *P* = 0.037) were independent risk factors associated with disease-free survival.

**Table 3:** Univariate and multivariate analyses for disease-free survival

Variables	Univariate analysis			Multivariate analysis		
	HR	95% CI	P	HR	95% CI	*P*
Age, years	1.204	0.735–1.975	0.461			
Sex	0.879	0.413–1.870	0.738			
AFP	1.186	0.695–2.025	0.531			
Cirrhosis	0.604	0.328–1.114	0.106			
Tumor size, cm	1.117	0.604–2.068	0.723			
Differentiation	1.135	0.604–2.132	0.694			
TNM stage	0.783	0.486–1.261	0.314			
Vascular invasion	1.727	1.049–3.117	0.024	1.562	1.013–2.815	0.042
HBV infection	1.763	1.069–2.906	0.026	1.650	1.017–2.676	0.037

Abbreviations: *HBV* hepatitis B virus

## Discussion

HBV infection is the main cause of HCC [1, 2]. But the mechanisms of carcinogenesis are different compared with NBNC-HCC [20, 22]. HBV is a DNA virus that causes malignant transformation of hepatocytes mainly through gene integration into chromosomes and activation of oncogenes [25–28]. The mechanism of NBNC-HCC is still unclear, and metabolic factors may be an important factor [21, 22]. For example, persistent non-alcoholic steatohepatitis can also cause sustained proliferation and repair of hepatocytes [29, 30]. During this process, hepatocytes undergo malignant transformation and eventually lead to the occurrence of HCC. Different carcinogenic pathways may lead to differences in the clinicopathological features and prognosis of HCC. Our study concluded that NBNC-HCC are significantly different from HBV-HCC in clinical features. In our study, we found that patients with HBV-HCC were younger, while patients with NBNC-HCC were older. The study also found that HBV-HCC has a higher proportion of vascular invasion, which may involve certain carcinogenic factors of HBV, such as increased cell motility caused by HBxAg [31–34]. However, there are still many gaps to be filled.

Massimo found that patients with HBV-HCC are younger than HCV-HCC patients [35]. The data from our study confirmed that patients with HBV-HCC were significantly younger than NBNC-HCC. Moreover, our study found that patients with HBV-HCC were more prone to vascular invasion. The prognosis of HBV-HCC was also worse than that of NBNC-HCC. TNM staging and vascular invasion are also factors influencing the long-term survival of patients with HCC.

There have been some studies comparing the prognostic differences between virus-associated HCC and NBNC-HCC [22–24]. Cescon et al. [36] reported that patients with NBNC-HCC have a significantly better relapse free survival rate than patients with HBV-HCC. Other studies have indicated that the survival rate of patients with NBNC-HCC was significantly higher than that of patients with HCV-HCC [37, 38]. Another study has shown that relapse free survival in patients with NBNC-HCC is significantly better than that in patients with HBV-HCC [23]. Our study confirmed that the prognosis of NBNC-HCC patients was superior to that of HBV-HCC patients, in terms of overall survival and disease free survival.

The relationship between prognosis and viral status in HCC patients is controversial. Kondo et al. [37] reported that the prognosis of NBNC-HCC patients is significantly better than that of HBC-HCC and HCV-HCC patients. Another study [39] suggested that the prognosis of nonalcoholic fatty liver disease related HCC was significantly better than that of HCV-HCC patients. However, Akahoshi et al. [40] suggested that there is no difference in prognosis between NBNC-HCC and other type of HCCs. This may be because the etiology of NBNC-HCC is not clear, and includes metabolic reasons, or alcohol abuse. In our study, we showed that NBNC-HCC patients have a better prognosis than HBV-HCC patients. These results provide further evidence for the prognosis of NBNC-HCC.

Some studies have evaluated prognosis after hepatic resection for HCC patients with different etiologies [41, 42]. Cescon et al. [36] reported that NBNC-HCC patients had significantly better outcomes than HBV-HCC patients did. In our study, we confirmed that the prognosis of NBNC-HCC patients was significantly better than that of HBV-HCC patients. We further found that in patients with HCC, TNM stage and HBV infection were independent prognostic variables for overall survival, while vascular invasion and HBV infection were independent risk factors associated with disease-free survival.

Although serum viral markers are important for HCC screening, ethnicity is another important factor [43]. A study has suggested that ethnicity plays an important role in the diagnosis and treatment of HCC [44]. In addition, the onset of HCC occurs at a median age of 45 in sub-Saharan African people, whereas a mean age of 52 to 65 has been observed in the rest of the world [45, 46]. In our study, the patients enrolled were all Asians and therefore ethnicity cannot be used as a variable. Further studies are needed to evaluate the differences in prognosis between different ethnic groups of HBV-HCC and NBNC-HCC patients.

There were some limitations in this study. First, the sample size was relatively small, so the results may be biased. The data collected in this study came from a single center and may lead to some enrollment bias; therefore, a multicenter prospective study is needed to further validate the results. Thirdly, since HCV-infected patients are relatively fewer in China, HCV-HCC patients were not enrolled in this study.

In summary, the clinicopathological features and prognosis of HBV-HCC and NBNC-HCC are significantly different. HBC-HCC is more common in young males with vascular invasion, while NBNC-HCC occurs mostly in elderly patients, and overall survival rate is significantly higher than that of HBV-HCC patients.

## Supplementary information

**Additional file 1.** The flow chart of the study is shown.

## Data Availability

Authors can confirm all relevant data are included in the article and materials are available on request from the authors.
