# Supplementary material for: Comparison of clinical features and outcomes between HBV-related and non-B non-C hepatocellular carcinoma
Source: Infect Agent Cancer. 2020 Feb 14;15:11. doi: 10.1186/s13027-020-0273-2 (PMC7023697; doi:10.1186/s13027-020-0273-2)
Supplement: Supplementary file 1 — Additional file 1. The flow chart of the study is shown. [file 13027_2020_273_MOESM1_ESM.docx]

A total of 226 HCC patients

137 patients with HBsAg(+) and HCV-Ab(-)

77 patients with HBsAg(-) and HCV-Ab(-)

A total of 107 patients enrolled and assigned to HBV-HCC group.

Exclude due to:

- Additional cancer(n=2)
- Incomplete data (n=28)

Exclude due to:

- Additional cancer(n=1)
- Incomplete data (n=8)

A total of 68 patients enrolled and assigned to NBNC-HCC group.

.

12 patients with HCV-Ab (+) and excluded
